# *Glycyrrhiza* Genus: Enlightening Phytochemical Components for Pharmacological and Health-Promoting Abilities

**DOI:** 10.1155/2021/7571132

**Published:** 2021-07-24

**Authors:** Javad Sharifi-Rad, Cristina Quispe, Jesús Herrera-Bravo, Lisandra Herrera Belén, Rajandeep Kaur, Dorota Kregiel, Yadav Uprety, Ahmet Beyatli, Balakyz Yeskaliyeva, Celale Kırkın, Beraat Özçelik, Surjit Sen, Krishnendu Acharya, Farukh Sharopov, Natália Cruz-Martins, Manoj Kumar, Ahmad Faizal Abdull Razis, Usman Sunusi, Ramla Muhammad Kamal, Shabnum Shaheen, Hafiz Ansar Rasul Suleria

**Affiliations:** ^1^Phytochemistry Research Center, Shahid Beheshti University of Medical Sciences, Tehran, Iran; ^2^Facultad de Ciencias de la Salud, Universidad Arturo Prat, Avda. Arturo Prat 2120, Iquique 1110939, Chile; ^3^Departamento de Ciencias Básicas, Facultad de Ciencias, Universidad Santo Tomas, Chile; ^4^Center of Molecular Biology and Pharmacogenetics, Scientific and Technological Bioresource Nucleus, Universidad de La Frontera, Temuco 4811230, Chile; ^5^Department of Chemical Engineering, Universidad de La Frontera, Av. Francisco Salazar 01145, Box 54D, Temuco, Chile; ^6^Louis Riel School Division, Winnipeg, Manitoba, Canada R2M 3R3; ^7^Lodz University of Technology, Faculty of Biotechnology and Food Sciences, Wolczanska 171/173, Lodz 90-924, Poland; ^8^Research Centre for Applied Science and Technology (RECAST), Tribhuvan University, P.O. Box 1030, Kirtipur, Kathmandu, Nepal; ^9^University of Health Sciences, Department of Medicinal and Aromatic Plants, Istanbul 34668, Turkey; ^10^Al-Farabi Kazakh National University, Faculty of Chemistry and Chemical Technology, Almaty 050040, Kazakhstan; ^11^Department of Gastronomy and Culinary Arts, School of Applied Sciences, Ozyegin University, Cekmekoy, 34794 Istanbul, Turkey; ^12^Department Food Engineering, Faculty of Chemical and Metallurgical Engineering, Istanbul Technical University, Maslak, 34469 Istanbul, Turkey; ^13^BIOACTIVE Research & Innovation Food Manufacturing Industry Trade Ltd. Co., Maslak, Istanbul 34469, Turkey; ^14^Molecular and Applied Mycology and Plant Pathology Laboratory, Department of Botany, University of Calcutta, Kolkata 700019, India; ^15^Department of Botany, Fakir Chand College, Diamond Harbour, West Bengal 743331, India; ^16^Research Institution “Chinese-Tajik Innovation Center for Natural Products”, National Academy of Sciences of the Republic of Tajikistan, Ayni St. 299/2, Dushanbe 734063, Tajikistan; ^17^Faculty of Medicine, University of Porto, Alameda Prof. Hernâni Monteiro, 4200-319 Porto, Portugal; ^18^Institute for Research and Innovation in Health (i3S), University of Porto, 4200-135 Porto, Portugal; ^19^Institute of Research and Advanced Training in Health Sciences and Technologies, CESPU, Rua Central de Gandra 1317, 4585-116 Gandra, PRD, Portugal; ^20^Chemical and Biochemical Processing Division, ICAR-Central Institute for Research on Cotton Technology, Mumbai 400019, India; ^21^Department of Food Science, Faculty of Food Science and Technology, Universiti Putra Malaysia (UPM), 43400 Serdang, Selangor, Malaysia; ^22^Natural Medicines and Products Research Laboratory, Institute of Bioscience, Universiti Putra Malaysia (UPM), 43400 Serdang, Selangor, Malaysia; ^23^Department of Biochemistry, Bayero University Kano, PMB 3011 Kano, Nigeria; ^24^Department of Pharmacology, Federal University, PMB 7156 Dutse, Jigawa State, Nigeria; ^25^Department of Plant Sciences, LCWU, Lahore 54000, Pakistan; ^26^Department of Agriculture and Food Systems, The University of Melbourne, Melbourne 3010, Australia

## Abstract

The *Glycyrrhiza* genus, generally well-known as licorice, is broadly used for food and medicinal purposes around the globe. The genus encompasses a rich pool of bioactive molecules including triterpene saponins (e.g., glycyrrhizin) and flavonoids (e.g., liquiritigenin, liquiritin). This genus is being increasingly exploited for its biological effects such as antioxidant, antibacterial, antifungal, anti-inflammatory, antiproliferative, and cytotoxic activities. The species *Glycyrrhiza glabra* L. and the compound glycyrrhizin (glycyrrhizic acid) have been studied immensely for their effect on humans. The efficacy of the compound has been reported to be significantly higher on viral hepatitis and immune deficiency syndrome. This review provides up-to-date data on the most widely investigated *Glycyrrhiza* species for food and medicinal purposes, with special emphasis on secondary metabolites' composition and bioactive effects.

## 1. Introduction

**Figure 1:**
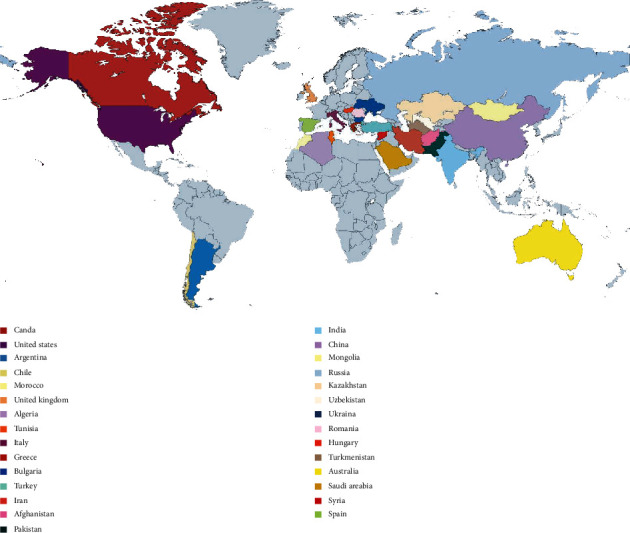
World map showing the major countries producing *Glycyrrhiza* spp.

The *Glycyrrhiza* genus belongs to the Fabaceae family and is generally well-known as licorice in English, *Bois doux* in French, and *Regalizia* in Spanish [1]. *Glycyrrhiza* is derived from the old Greek terms *glykos* which means “sweet” and *rhiza* which means “root” [2]. Composed of ~30 species, this genus is distributed worldwide [3], and is extensively spread in the Mediterranean basin of Africa, Europe, and Asia, extending to Australia, North America, and temperate regions of South America. *Glycyrrhiza glabra* L. is commercially the most important species under this genus, native to Afghanistan, Syria, Persia, Southern Europe, and North Africa [4, 5]. This species consist of 3 varieties, viz., *Glycyrrhiza glabra* var. *violacea*, known as Persian and Turkish licorice; *Glycyrrhiza glabra* var. *gladulifera*, known as Russian licorice; and *Glycyrrhiza glabra* var. *typica* L., known as Spanish and Italian licorice [6]. The second most commercially important species of this genus is *Glycyrrhiza uralensis* Fisch. which extends from Western to Eastern Siberia, as well as from Northern China to Mongolia [7]. *Glycyrrhiza echinata* L. is common in the Balkans and in Russia [8]. *Glycyrrhiza pallidiflora* Maxim. is native to the far East and China, *Glycyrrhiza asperrima* L.f. is commonly found in Russia and in Central Asia, *Glycyrrhiza astragalina* Hook. & Arn. is found Chile, and *Glycyrrhiza bucharica* Regel is found in Central Asia [8]. In Pakistan, the genus is represented by only 3 species, viz., *Glycyrrhiza glabra* L., *Glycyrrhiza triphylla* Fisch. & C.A.Mey., and *Glycyrrhiza uralensis* Fisch. [1, 9]. *Glycyrrhiza áspera* Pall., *Glycyrrhiza echinata* L., *Glycyrrhiza glabra* var. *glabra*, *Glycyrrhiza glabra* var. *glandulifera*, and the endemic species *Glycyrrhiza asymmetrica* Hub.-Mor., *Glycyrrhiza icónica* Hub.-Mor, and *Glycyrrhiza flavescens* subsp. *flavescens* grow among the Turkish flora [10–14]. Nonetheless, and looking at large-scale use, some taxa of the genus are now commercially cultivated in Spain, Sicily, England, North America, and Northern India [15–18]. The world map showing the countries which widely cultivate licorice can be seen in Figure 1.

**Figure 2:**
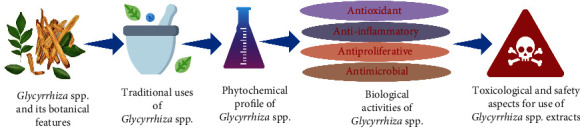
Diagram showing various components discussed in the review.

Licorice has certain bioactive components such as flavonoids and glycyrrhizin, which have many pharmacological properties such as antioxidant, antiviral, anti-infective, and anti-inflammatory properties [19, 20]. The spreading cultivation of the *Glycyrrhiza* spp. is due to its high market demands pertaining to their pharmaceutical properties. Traditionally, licorice is widely used as an antidote to reduce the toxicity caused by drug therapies. In Ayurveda, licorice is considered as “Rasayana,” which means to have nourishing, rejuvenating, and strengthening effects. Its rhizomes and roots are used to treat renal, hepatic, gastric, and respiratory disorders. Recent findings established its role in numerous biological activities in the human body such as having an anti-inflammatory and antioxidant role and having a protective effect on various organs [21]. It is evident that licorice has a various health-promoting activities proved using preclinical and clinical trials; however, a comprehensive compilation of these research advances is lacking. In this sense, the present work is aimed at providing an in-depth revision on the most widely used and investigated *Glycyrrhiza* spp. for food and medicinal purposes. A special emphasis is given to the botanical aspects, traditional uses, and secondary metabolite composition of widely studied species of *Glycyrrhiza* (*Glycyrrhiza glabra* L. and *Glycyrrhiza uralensis* Fisch.) and their biological effects. The basic components discussed in the current review are presented in Figure 2.

## 2. Botanical Features

The *Glycyrrhiza* genus is a subshrub, erect, and attaining heights of up to 2.5 m tall. It has highly developed stoloniferous roots. Roots are thick and branched with yellowish to red color [22]. Leaves are alternate, petiolate, and imparipinnate, with small, free, deciduous stipules; leaflets are elliptical to lanceolate, 4–7 pairs; apex acute to obtuse, margin entire, smooth [23, 24]. The inflorescence has an axillary spike, and bracts are very small, conspicuously present or absent, and caduceus. Flowers are stipitate, narrow, and zygomorphic. The calyx is short, persistent, and bilabiate. The corolla is typically papilionaceous and blue, lavender to purple, or violet in color; the banner petal (vexillum) is narrow or oblanceolate; the wing petals (alae) are narrow, oblanceolate to oblong; and the keel petal is bipartite, acute, and straight. Stamens are diadelphous, filaments are glabrous, and anthers are simple and rounded. Style is filiform, and stigma is blunt. Legume or pod is the fruit of *Glycyrrhiza* genus which is compressed, oblong, unilocular, up to 1.5 cm long, sometimes falcate, erect, glabrous or covered with brownish echinate glandular hairs (*Glycyrrhiza uralensis* Fisch.), and weakly dehiscent or indehiscent. Seeds are reniform; smooth; small; and brown, black, or deep grey [10, 22, 24, 25].

## 3. Traditional Uses

The *Glycyrrhiza* genus is used around the world for food and medicinal purposes [26]. The Generally Recognized as Safe (GRAS) status of licorice allows its application in a plethora of foods at typical concentrations. Also, the sweet flavor of licorice makes it suitable for various applications in foods, like confectionary and condiments, where the most commonly used plant part are the rhizomes and roots. For example, both London drops and Red Vines® are flavored with licorice. In condiments, licorice powder is generally used in sweet chili sauce and soy sauce to add a unique flavor. In traditional medicine and clinical practice of different cultures, *Glycyrrhiza* has been applied for treating various types of ailments [2]. Although there are more than 20 species identified belonging to this genus, just a few of these species are traditionally used to treat diseases.

The roots of *Glycyrrhiza glabra* L. are traditionally most commonly used in Albania and Italy against sore throat and as an antitussive [27]. In Brazil, *Glycyrrhiza glabra* L. is used as an emollient and diuretic, and it is also used for inflammatory diseases [28]. In the countries of the Commonwealth of Independent States, *Glycyrrhiza glabra* L. has been used as an expectorant, emollient, anti-inflammatory, antispasmodic, antacid, antiallergic, antihistamine, laxative, diaphoretic, analgesic, diuretic, wound-healing medicine, tonic, potency enhancer, detoxificant, sedative, antiviral, antiulcer, hypotensive medicine, capillary-strengthening medicine, antibacterial, and antioxidant [29, 30]. The *Glycyrrhiza glabra* L. rhizome is traditionally used in the Northern Navarra, Iberian Peninsula, for digestive disorders [31]. In India, *Glycyrrhiza glabra* L. barks are used by local traditional practitioners in the Thiruvarur district for gonorrhea, while the whole plant is used for hepatitis B virus (HBV) infection [32]. The whole ripe fruit and rhizome are used to increase the sperm count and to treat colds and cough [33]. Fruit and stem bark of this species are also used for paralysis [34]. Wild grown roots and leaves are used in different parts of Iran for gastralgia, gastric ulcer, hemorrhoids, liver disorders, muscle spasm, bone pains, and dyspnea [35, 36]. In the Kerman province of Iran, the roots and stems are used by local people for colds, stomach pain, ulcers, acidification, joint pain (back and leg), and bone fractures [37, 38]. In the Qaysari Market, Erbil, Iraq, *Glycyrrhiza glabra* L. radix is applied for pneumonia, sour eructation, and duodenal inflammation [39]. *Glycyrrhiza glabra* L. is traditionally used to treat sore throat and lung infections in Mauritius [40]. In South Africa, local people often use *Glycyrrhiza glabra* L. rhizomes for chest ailments, rheumatism, arthritis, and ulcers [41, 42]. *Glycyrrhiza glabra* L. rhizomes are used for common colds in the Granada province of Southern Spain [43]. The roots of this plant are used for the treatment of heart diseases by Turkish communities in Germany [44]. Also, roots are used by the local people in Turkey for respiratory tract diseases, flu, bronchitis, pain, epilepsy, cancer, gastrointestinal diseases, and high cholesterol levels [45–47]. Besides, roots are used for cardiac disorders, diabetes, and hemorrhoids [48, 49].

Among other *Glycyrrhiza* spp., *Glycyrrhiza echinata* L. is used in West Azerbaijan (Iran) in cases of cough, bronchitis, ulcer, and pharynx [50]. Also, the underground parts of *Glycyrrhiza uralensis* Fisch. are used in Kazakhstan as an anticoagulant, antifibrinolytic, diuretic, anti-inflammatory, and detoxificant, as well as for handling diabetes mellitus and hypertension [51]. The use of the licorice extracts is also mentioned in traditional Chinese medicine. It was recommended for relieving the situation of dyspnea, spasms, cough, phlegm, pain, and toxicity as traditional Chinese medicine. Kampo, which is a traditional Japanese medicine also named as “yokukansan,” shows a neuroprotective effect, whereas in South Korea it has been used in the treatment of cold and cough. It is conspicuous that rhizomes and roots from licorice are used widely in many countries as traditional medicine, and it may act as an important ingredient in the formulation of functional foods.

The medicinal properties of this plant are mainly conferred by the phytoconstituents, and it is crucial to know the exact profile. The next section will highlight the phytochemical profile of licorice.

## 4. Secondary Metabolite Composition

**Figure 3:**
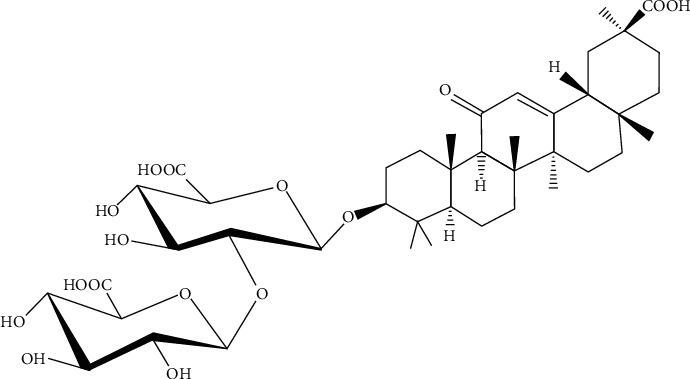
Glycyrrhizin (glycyrrhizinic acid) structure.

As licorice represents a historical background of use, and various investigations have been performed to find the active principles responsible for the extreme health potentialities. Glycyrrhizin is the principal substance present in licorice roots (Figure 3), along with the flavonoid liquiritin apioside [52, 53]. Four compounds were isolated including two new flavonoids from the licorice of Sinkiang, China. The known compounds were licochalcones A and B. A new compound, glycyrrhisoflavanone, was obtained as colorless needles. The electron ionization mass spectrometry (EIMS) of both compounds showed the molecular ion peaks at 368 and 354, which correspond to the molecular formulas C_21_H_20_O_6_ and C_20_H_18_O_6_, respectively [54].

Phenolic compounds, such as liquiritigenin, isoliquiritigenin, 4′-O-methylglabridin, isoprenyl chalcone, formononetin, glabridin, and hispaglabridins A and B are also present at greater amounts and possess numerous bioactivities [55–59]. Stilbene derivatives have also been isolated from licorice leaves [60, 61]. Moreover, 49 phenolic compounds and 15 different saponins have been identified from licorice roots [62]. Saponins (licorices A3, G2, and J2), chalcones (isoliquiritin, licochalcone B, and neolicuroside) [63], coumarins (glycycoumarin) [64], and flavonoids (glychionides A and B, glabrene, glabrone, glabraisoflavanones A and B, isoviolanthin, 5,7-dihydroxyflavanone, and rhamnoliquiritin) [24, 65–67] have also been reported in licorice. Glucoliquiritin apioside, prenyl licoflavone A, shinflavanone, shinpterocarpin, and 1-methoxy phaseolin are also present in licorice roots [68]. The optimization of the extraction of glycyrrhizic acid and glabridin from Chinese licorice was investigated [69]. In another study, it was found that the main constituents of *Glycyrrhiza glabra* L., *Glycyrrhiza uralensis* Fisch., and *Glycyrrhiza inflata* Batalin were glycyrrhizin, liquiritin and its apioside, and liquiritigenin [53]. A brief description of characteristic compounds present in some of the important species of licorice is also presented below.

Regarding *Glycyrrhiza* spp. essential oils, a remarkable difference has been found in volatile constitution. Volatile compounds, such as linalool oxides A and B, terpinen-4-ol, *α*-terpineol, and geraniol, have been identified from roots [70].

### 4.1. *Glycyrrhiza glabra* L.

The methanol based-root extract of *Glycyrrhiza glabra* L. was partitioned between water and ethyl acetate to obtain water and ethyl acetate soluble fractions. These fractions were then separated using column chromatography and yielded 3 known compounds, viz., kanzonol Y, licochalcone C, licoagrochalcone B, and one new compound, named glycyglabrone [71]. Another group of researchers found the presence of 15 sugars in the ethanol extract of *Glycyrrhiza glabra* L., with ribitol, saccharose, glucofuranose, sorbose, fructose, mannitol, galactofuranose, mannopyranose, hydroxyethylglucose, glucopyranose, and mannopyranosyl-D-glucitol being the most common [72]. The same authors separated the components of *Glycyrrhiza glabra* L. roots based on acid-base properties. Glabridin, hispaglabridin B, and 4-O-methylglabridin were the major compounds of the fraction identified by gas chromatography-mass spectrometry (GC-MS) [73].

**Figure 4:**
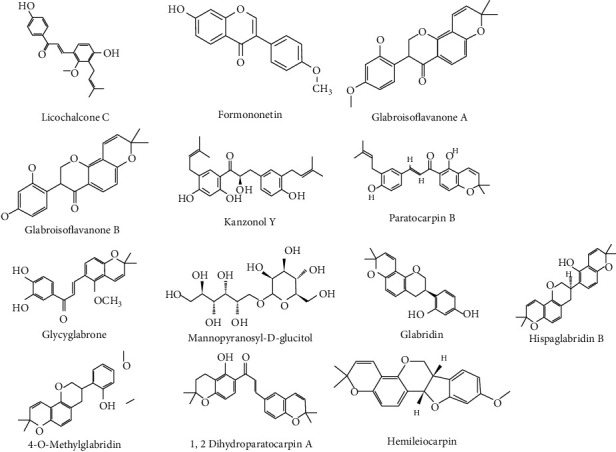
Chemical structures of the main components reported in *Glycyrrhiza glabra* L.

Two new compounds, viz., 1,2 dihydroparatocarpin A and neolignan lipid esters, along with 7 known phenolic compounds, viz., paratocarpin B, formononetin, isoliquiritigenin, glabridin, 4-O-methylglabridin, hemileiocarpin, and hispaglabridin B were discovered from the chloroform extract of *Glycyrrhiza glabra* L. roots and stolons [55]. In addition, three new compounds, namely, glabroisoflavanones A and B and glabrocoumarin were isolated from the dichloromethane extract of *Glycyrrhiza glabra* L. roots [74]. Ten monodesmosidic saponins were isolated, namely, 20-*α*-rhaoglycyrrhizin, 20-*α*-galacturonoylglycyrrhizin, 11-deoxo-20-*α*-glycyrrhizin, rhaogalactoglycyrrhizin, rhaoglucoglycyrrhizin, 11-deoxorhaoglycyrrhizin, rhaoglycyrrhizin, 24-hydroxyglucoglycyrrhizin, glycyrrhizin 20-methanoate, and 30-hydroxyglycyrrhizin were isolated for the first time using the aquamethanolic extract of *Glycyrrhiza glabra* L. roots [75]. The main components found in *Glycyrrhiza glabra* L. are represented in Figure 4.

### 4.2. *Glycyrrhiza uralensis* Fisch.

Flavonoid glycosides (liquiritin, isoliquiritin, neoisoliquiritin, ononin, liquiritin apioside, isoliquiritin apioside, and licraside) and triterpene glycosides (glycyrrhizin; araboglycyrrhizin; 18 *α*-glycyrrhizin; apioglycyrrhizin; and licorice saponins A3, E2, G2, H2, and L3) were extracted from the *Glycyrrhiza uralensis* Fisch. roots [76]. Echinatin, licoflavone A, licochalcone A, liquiritin, formononetin, glabrono, licochalcone B, isoliquiritin, 4,7-dihydroxy-flavone, liquiritigenin, and medicarpin 3-O-*β*-D-glucopyranoside were also identified in *Glycyrrhiza uralensis* Fisch. [77]. Other researchers also found licochalcone B in the ethanolic extract from *Glycyrrhiza uralensis* Fisch. roots [78]. Isoliquiritigenin, its glycoside isoliquiritin, and isoliquiritin apioside forms were also isolated from *Glycyrrhiza uralensis* Fisch. aqueous extract [79]. Hayashi et al. [80] also isolated a triterpene saponin, glucoglycyrrhizin, from *Glycyrrhiza uralensis* Fisch.

**Figure 5:**
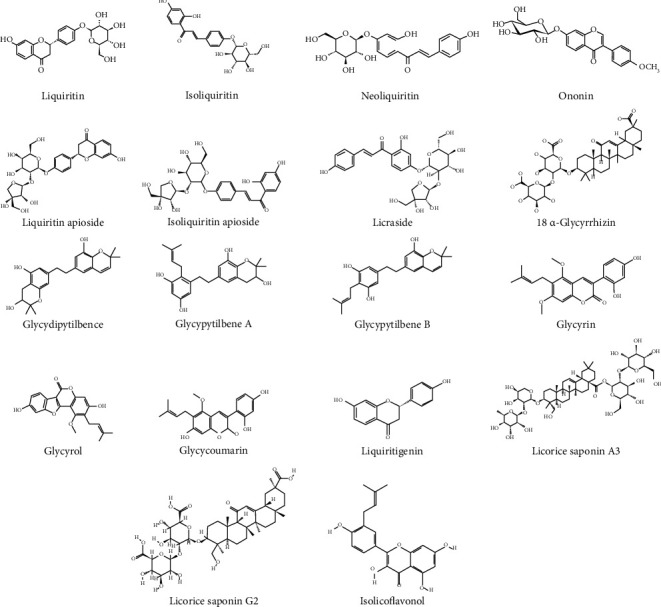
Chemical structures of the main components reported in *Glycyrrhiza uralensis* Fisch.

Three novel dihydrostilbenes, particularly glycydipytilbene and glycypytilbenes A and B with 12 known compounds, were isolated from *Glycyrrhiza uralensis* Fisch. leaves. The compounds were identified as isoglycyrol, ononin, glycycoumarin, glycyrrhizic acid, licuraside, liquiritin, isoliquiritin apioside, liquorice saponin A3, licorice glycosides A and B, liquiritin apioside, licorice saponin G2, and isolicoflavonol [81]. In another research, thirty-four known compounds and 2 new compounds were identified from the ethanolic extract of *Glycyrrhiza uralensis* Fisch. roots [82]. In another work, glycyrin, glycyrol, glycycoumarin, and liquiritigenin were also obtained from the dried powder of *Glycyrrhiza uralensis* Fisch. roots [83]. The main components found in *Glycyrrhiza uralensis* Fisch. are represented in Figure 5.

### 4.3. Other *Glycyrrhiza* Species

Three pure compounds, namely, glepidotins A, B, and D were identified from *Glycyrrhiza lepidota* Pursh leaves. These compounds were extracted by a solvent system containing H_2_O, and CH_2_Cl_2_ : MeOH in a 1 : 1 ratio. The identification was done using bioassay-guided fractionation and high-performance liquid chromatography (HPLC) [84].

A new retrochalcone and three known compounds were isolated from the roots of *Glycyrrhiza inflata* Batalin. The structure of retrochalcone and licochalcone E were elucidated through spectroscopic analysis. Isoliquiritigenin, licochalcone A, and licochalcone C were also identified by analysis of the mass spectrometry (MS), nuclear magnetic resonance (NMR), and infrared spectroscopy (IR) spectra of each compound and by comparing with those found in the literature [85].

It is evident that 4 species under the *Glycyrrhiza* genus, namely, *Glycyrrhiza glabra* L., *Glycyrrhiza uralensis* Fisch., *Glycyrrhiza lepidota* Pursh, and *Glycyrrhiza inflata* Batalin, are widely studied for the investigation of bioactive compounds, and these compounds may reveal important use as health-promoting agents.

## 5. Biological Activities

The biological effects of the *Glycyrrhiza* genus have been mainly assessed through *in vivo* and *in vitro* experiments. *Glycyrrhiza* plant extracts have been majorly assessed for its antioxidant, antimicrobial, anti-inflammatory, antiproliferative, and cytotoxic activities. Nevertheless, the observed biological activities of *Glycyrrhiza* can vary according to the extraction method [86], geographical origin [87, 88], drying method [89], and harvesting time [90].

### 5.1. Antioxidant Activity

The antioxidant potential of *Glycyrrhiza* spp. has been reported by several studies [91–98] as assessed through its *in vitro* radical scavenging potential, phosphomolybdenum, cupric-reducing antioxidant capacity (CUPRAC), 2,2-diphenyl-1-picrylhydrazyl (DPPH) assay, hydrogen peroxide scavenging capability, and *β*-carotene/linoleic acid bleaching assays. Shakeri et al. [99] assessed the biological activity of *Glycyrrhiza triphylla* Fisch. essential oils and found an appropriate antioxidant activity with an IC_50_ of 110.4 *μ*g/mL using the DPPH assay. Polysaccharide fractions (GUPs-1, GUPs-2, and GUPs-3) extracted from *Glycyrrhiza uralensis* Fisch. were reported to have antioxidant effects [100]. Among all the fractions, GUPs-1 demonstrated the highest scavenging activity (70%) at a concentration of 4 mg/mL followed by GUPs-2 (60%) and GUPs-3 (30%). Licorice phenolic extract at a concentration of 0.54 *μ*M was revealed to have the highest oxidative stress protection with 72% of cell viability in Caco-2 cells [95]. Haraguchi et al. [101] reported that isoflavans extracted from *Glycyrrhiza glabra* L. inhibited oxidative stress in liver mitochondria. The most potent inhibitor of NADH-dependent lipid peroxidation was 3′-hydroxy-4′-O-methylglabridin with an IC_50_ of 0.1 *μ*M. The high *in vitro* antioxidant capacity and inhibitory effect of *Glycyrrhiza glabra* L. extracts on peroxidation of lipids in mice liver was also reported by Saeed et al. [102]. Furthermore, the oral intake of *Glycyrrhiza glabra* L. polysaccharides led to an improvement in immune system activity and decreased oxidative stress in high-fat mice by enhancing the activity of the antioxidant enzymes [103]. A dose of 300 mg/kg of *Glycyrrhiza glabra* polysaccharides resulted in the highest activity of superoxide dismutase (150 U/mL), catalase (3.33 U/mL), and glutathione peroxidase (20.67 U/mL). On the other hand, dehydroglyasperins C and D and isoangustone A separated from *Glycyrrhiza uralensis* Fisch. inhibited lipid peroxidation with IC_50_ values varying between 0.205 and 0.418 mM in rat tissue. Further, 10 *μ*M licorice extracts also inhibited H_2_O_2_-induced reactive oxygen species (ROS) production by 53–85% in human hepatoma (HepG2) cells [104]. The level of ROS induced by *Glycyrrhiza glabra* L. root infusions was also reported to have a good mineral content and *in vitro* antioxidant activity with 52% of OH scavenging ability. The extracts did not cause degenerative effects up to 50 mg/kg b.w. in rat hepatocytes (Salawu, Ibukun, and Esan, 2019 [105]). Furthermore, Liu et al. [106] isolated eight new triterpenoid saponins from *Glycyrrhiza uralensis* Fisch. and observed that two compounds, glyuralsaponins B and H, displayed lipid peroxidation inhibition activity against Fe^2+^/cysteine-induced liver microsomal enzyme system at a concentration of 0.1 *μ*M with inhibition values of 79 and 91%, respectively.

Besides these aspects, licorice has also revealed a good ability to be used as a natural antioxidant in food products. For instance, licorice extract was effective in controlling rancidity in precooked pork [56]. The value of thiobarbituric acid-reactive substances in the control sample of pork patties was found to be 9.4 mg/kg after 14 days of storage, whereas it was only 4.4 mg/kg in the case of licorice extract-treated pork patties. Furthermore, its antioxidant activity in fish oil was also assessed by various methods, as reported by Ucak [107]. On the other hand, Zhang et al. [108] recommended licorice extract to be fed to sheep as a bioadditive to enhance the antioxidant effect of their meats. Compared to the control samples, the supplementation of 3000–4000 mg/kg feed led to increased DPPH (39%) free radical scavenging activity.

### 5.2. Anti-Inflammatory Activity

The methanol extract from leaves of *Glycyrrhiza glabra* L. and *Glycyrrhiza uralensis* Fisch. demonstrated anti-inflammatory activity on lipopolysaccharide- (LPS-) induced RAW264.7 cells [109–112]. Moreover, Frattaruolo et al. [113] reported that the M2 fraction (licoflavanone) from *Glycyrrhiza glabra* L. leaves at an IC_50_ value of 60.49 *μ*M exhibited anti-inflammatory activity by reducing the NF-*κ*B translocation as confirmed by using immune-fluorescence monitoring and reducing the nitrite levels by ~100-fold while reducing the proinflammatory cytokines and cyclooxygenase 2/inducible nitric oxide synthase expression. The anti-inflammatory activity of *Glycyrrhiza uralensis* Fisch. was also reported [114]. The authors reported that acetone licorice fractions can act as a strong anti-inflammatory agent and caused 77.9% inhibition at 62 *μ*g/mL.

Regarding the *in vivo* findings, licorice flavonoids at a concentration of 30 mg/kg revealed anti-inflammatory effects by reducing the expression of TNF*α* and IL-1*β* mRNA expression on mice with acute pulmonary inflammation and licochalcone A at a concentration of 20 *μ*M also revealed anti-inflammatory effects on mice with acute lung injury, as demonstrated by Me et al. [115] and Chu et al. [116], respectively. Liu et al. [117] also reported that licochalcone A of licorice roots has anti-inflammatory activity in mice, while Khattab et al. [118] reported that 10 mg/kg of licorice extracts demonstrated anti-inflammatory activities in mice with ovalbumin-induced bronchial asthma by reducing the levels of interleukin- (IL-) 5 and 13 and IgE.

### 5.3. Antiproliferative and Cytotoxic Activity

*Glycyrrhiza glabra* L. methanolic extract demonstrated interesting antiproliferative effects in a skin cancer melanoma cell line (WM1316A) at an IC_50_ value of 35.2 *μ*g/mL [119]. Vlaisavljević et al. [120] evaluated the chemical composition of licorice root extracts and reported that fresh root extracts had antiproliferative activity against human cancer cell lines of gynecological origin containing four breast lines (T47D, MCF7, MDA-MB-231, and MDA-MB-361), two cervical cancer cell lines (HeLa and SiHa), and one ovarian cancer cell line (A2780). The authors reported that 30 *μ*g/mL fresh root extracts of licorice demonstrated >50% growth inhibition in all the cell lines except HeLa. In addition, Jo et al. [121] also concluded that *Glycyrrhiza glabra* L. roots exhibited preventive activity against breast cancer. *Glycyrrhiza glabra* L. extract also demonstrated cytotoxicity against hepatocellular, breast, and colorectal cancer cell lines with IC_50_ values in the range of 5.6 to 33.6 *μ*g/mL [122]. It was also revealed that the cytotoxic activity of the methanol extract of licorice roots against immortal human keratinocyte, lung adenocarcinoma, and liver carcinoma cell lines varied depending on geographical origin [123].

Rasul and Ma [124] reported the cytotoxic activity of *Glycyrrhiza uralensis* Fisch. against the gastric adenocarcinoma (SGC-7901) cell line with IC_50_ values in the range of 8.7 to 64.9 *μ*g/mL for different compounds. Moreover, Fan et al. [125] identified the biologically active components of *Glycyrrhiza uralensis* Fisch. leaves and noted that most of these compounds exhibited an antiproliferative effect on human hepatic stellate cells with IC_50_ values in the range of 43 to more than 90 *μ*g/mL for different compounds. *Glycyrrhiza pallidiflora* Maxim. also showed cytotoxicity on human T-cell leukemia (MT-4), human monocyte (U-937), and lymphoblastoid leukemia (CEM-13) cells [126]. The authors reported that isoflavonoid calycosin demonstrated the best results against human T-cell leukaemia MT-4 cells (cell toxicity dose (CTD)_50_, 2.9 *μ*M). Furthermore, the protein extract of licorice roots at concentrations of 50 and 100 *μ*g/mL revealed inhibitory effects and induced apoptosis of colon cancer cells [127]. In another study, it was claimed that glycyrrhetic acid induced apoptosis in non-small-cell lung cancer cell lines [128].

Regarding the *in vivo* findings, licorice extract has shown antitumor activity in mice with colon cancer [129]. Moreover, Liu et al. [117] reported that licochalcone A of licorice root has cytoprotective activity in mice.

### 5.4. Antimicrobial Effects

Several studies have reported the antimicrobial capacity of *Glycyrrhiza* spp. against several pathogens. *Glycyrrhiza glabra* L. extract showed interesting antimicrobial effects against several microorganisms, such as *Escherichia coli*, *Staphylococcus aureus*, *Pseudomonas fluorescens*, *Bacillus cereus*, *B. subtilis*, *Enterococcus faecalis*, *Candida albicans*, *C. glabrata*, and *Aspergillus niger* [97, 130–134]. Martins et al. [135] also evaluated the antimicrobial potential using the disc diffusion method and the antibiofilm activity of *Glycyrrhiza glabra* L. against *Candida* strains. The authors concluded that *Candida tropicalis* was most susceptible with a disc diffusion diameter of 10-13 mm. Furthermore, the antimicrobial capacity of licorice roots against *Helicobacter pylori* was reported by Nariman et al. [136]. The minimum inhibitory concentration (MIC) of the licorice root extract ranged between 15.6 and 250 *μ*g/mL.

Similarly, the *Glycyrrhiza uralensis* Fisch. ethanolic extract was also able to inhibit *E. coli*, *B. cereus*, and *S. aureus* with a diagram of zones of inhibition of 18, 14, and 12.4 mm, respectively [137]. *Glycyrrhiza uralensis* Fisch. hexane fraction also demonstrated a good antimicrobial effect with an MIC of 0.25 mg/mL against methicillin-resistant *S. aureus*, whereas the chloroform fraction had a 2.5-fold higher antimicrobial activity [138]. Chouitah et al. [139] also reported interesting antibacterial effects of the *Glycyrrhiza glabra* L. essential oil against *E. coli*, *S. typhi*, and *S. aureus* with an MIC of 4.2, 14.5, and 14.5 *μ*g/mL, respectively. It was reported that iconisoflavan, (3S)-licoricidin, licorisoflavan A, and topazolin exhibited antibacterial activity against *Salmonella typhimurium* [140]. Moreover, glycyrrhizin isolated from *Glycyrrhiza glabra* L. exerted antimicrobial activity against *S. sciuri*, *E. coli*, *S. typhi*, *S. aureus*, *Rhizopus* ssp., and *Aspergillus awamorii* with an inhibition diameter of 25, 28, 20, 25, 14, and 12 mm, respectively [98]. In addition, Chidambaram and Swaminathan [141] claimed that *Glycyrrhiza glabra* L. and glycyrrhizin exhibit antimicrobial activity against *Mycobacterium tuberculosis* with an MIC value of 500 *μ*g/mL. Similarly, the *Glycyrrhiza glabra* L. extract was revealed to be effective against *M. tuberculosis* in U937 human macrophage cell lines with an MIC varying from 0.97 to 1.95 *μ*g/mL [142]. Saponins from *Glycyrrhiza glabra* L. showed good antimicrobial potential on *E. coli*, *S. aureus*, and *P. aeruginosa* with an MIC of 3.12 mg/mL, whereas the MIC was 25 mg/mL for *Proteus mirabilis* strains [143]. An isolated compound from *Glycyrrhiza glabra* L., 18-*β*-glycyrrhetinic acid, was also effective against *C. albicans* isolated from patients with vulvovaginal candidiasis [144]. The authors reported that a concentration of 6.2 *μ*g/mL of 18-*β*-glycyrrhetinic acid can inhibit the growth of *C. albicans*. In another *in vivo* investigation, the *Glycyrrhiza glabra* L. extract (80 mg/kg dose) was significantly active for *P. aeruginosa* in mice with lung infection [145]. The authors reported that bacterial load increased constantly in control animals but was reduced by 3 log_10_ CFU/mL after 7 days of treatment with licorice extract. Moreover, Bawankule et al. [146] reported that CIM-Candy, prepared with *Ocimum sanctum* L., *Phyllanthus emblica* L., and *Glycyrrhiza glabra* L. according to Ayurvedic techniques, demonstrated humoral immune response in a mouse model.

Phytochemical constituents particularly licoricidin, glycyrin, and glycycoumarin were isolated from *Glycyrrhiza uralensis* Fisch., and licoricidin demonstrated the highest antibacterial activity against respiratory infection-associated bacteria, specifically *S. pyogenes*, *Haemophilus influenza*, and *Moraxella catarrhalis* with an MIC of 12.5 *μ*g/mL [147]. In addition, 1-methoxyficifolinol, licorisoflavan A, and 6,8-diprenylgenistein extracted from *Glycyrrhiza uralensis* Fisch. extract was also effective in preventing *S. mutans* biofilm formation [148]. On the other hand, Rajput et al. [149] reported the antimicrobial potential of the *Glycyrrhiza uralensis* Fisch. extract against the plant pathogens *Rhizoctonia solani* and *Pythium aphanidermatum*.

Licochalcones A-E isolated from *Glycyrrhiza inflata* Batalin demonstrated antibacterial activity against methicillin-resistant *S. aureus* and vancomycin-resistant *Enterococci* with the MIC ranging from 10 to 20 *μ*g/mL [150]. In addition, Tsukiyama et al. [151] reported a good antimicrobial activity to licochalcone A isolated from *Glycyrrhiza inflata* Batalin against several spore-forming bacteria. It was also reported that phenolic compounds isolated from licorice, such as licoarylcoumarin, glycycoumarin, and gancaonin I, showed potent or moderate antimicrobial activity against vancomycin-resistant *Enterococcus* with the MIC varying from 8 to 16 *μ*g/mL [152]. In another study, Celik and Duran [153] claimed that glycyrrhizinic acid exhibited antimicrobial activity against *H. pylori* strains. At the same time, Lv et al. [154] stated that *Glycyrrhiza* spp. could be used as an antimicrobial in cotton fabric.

**Figure 6:**
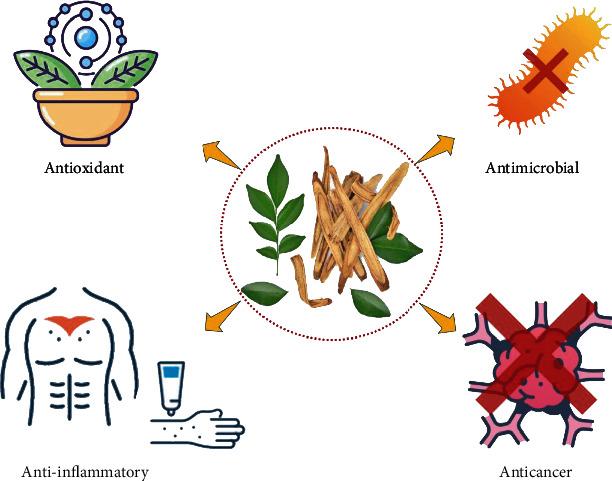
Various biological activities of the *Glycyrrhiza* species extract discussed in this study are presented in the diagram.

It is undisputable from the bioactivities (antioxidative, antimicrobial, antiproliferative, and anti-inflammatory) (Figure 6) of the extracts that licorice is certainly a potential candidate for wider application in functional foods and in the pharmaceutical industry for improving the health standards in humans. These health-promoting effects of the licorice extracts are also evident from the clinical studies presented in the next section.

## 6. Health-Promoting Effects: From Preclinical to Clinical Evidence

The pharmacological effects of various *Glycyrrhiza* spp.-derived bioactivities have been subject to several clinical studies in humans. Neo Miniphogen-C and glycyrrhizin are the most widely experimented derivatives in humans, and the efficacy has been reported to be significantly high in viral hepatitis and immune deficiency syndrome.

In 1993, Acharya et al. studied the activity of the Stronger Neo Minophagen-C (SNMC) interferon stimulator derived from *Glycyrrhiza glabra* L. at a dose of 40 or 100 mL daily for 8 weeks in 18 hepatitis patients, and a survival rate of 72.2% was obtained (*p* < 0.01). Moreover, SNMC demonstrated an anti-inflammatory activity in patients with liver disease [155]. In another study, an improvement in liver histology was reported by using SNMC, containing 40 mg glycyrrhizin, injected to patients with chronic viral hepatitis [156]. Glycyrrhizin also induced a decrease of 1.5-fold in serum liver enzymes and improved liver histology when intravenously administered [156, 157]. A similar finding on glycyrrhizin has also suggested that it may enhance liver function with occasional complete recovery [158–160].

A retrospective research was carried out to assess the ability of SNMC to prevent chronic hepatitis C (CHC) development, and it was found that long-term SNMC administration has proven to be effective [161]. In addition, SNMC could inhibit liver necrosis and inflammation in CHC patients [162]. Other authors also showed that glycyrrhizin notably reduces the hepatocarcinogenesis rate [163].

Glycyrrhizin derived from *Glycyrrhiza* spp. was tested intravenously at 400–1600 mg/daily for a period of more than one month to three hemophiliacs with acquired immune deficiency syndrome (AIDS), with glycyrrhizin being able to inhibit the human immunodeficiency virus- (HIV-) 1 replication *in vivo* [164, 165]. CD4 lymphocyte count and cluster of differentiation (CD)4/CD8 ratio increased by drip infusion of SNMC (5 mg glycyrrhizin/kg) in AIDS patients [166]. In another research, glycyrrhizin administration to HIV-positive hemophilia patients showed preventive effects by raising the CD4-positive T-lymphocyte number [167]. Also, the application of glycyrrhizinic acid for 12 months produced a positive effect with a 30–40% rate of success in patients with chronic viral hepatitis B [168].

Compared to other species of *Glycyrrhiza*, the *Glycyrrhiza glabra* L. aqueous extract was found to be active in reducing oral mucositis in cancer patients [169]. Another clinical trial suggested that an ImmunoGuard® drug—a standardized fixed herbal combination of *Glycyrrhiza glabra* L. with *Andrographis paniculata* (Burm.f.) Nees, *Eleutherococcus senticosus* (Rupr. & Maxim.) Maxim., and *Schisandra chinensis* (Turcz.) Baill. extracts—was safe and effective in Familial Mediterranean Fever (FMF) patient management [170].

In another clinical trial conducted with 236 patients (randomized and double-blind study), licorice-based gargles were found effective in postoperative sore throat and postextubation coughing as compared to sugar water-based gargles. The incidence of sore throat in the case of licorice-based gargles was 19% after a half-hour and only 10% after one hour of surgery, whereas incidence was, respectively, 36 and 35% in the case of sugar water-based gargles [171]. Ghalayani et al. [172] compared a licorice mucoadhesive film with a triamcinolone acetonide film on radiotherapy-induced oral mucositis in a randomized double-blind trial with 60 patients (*n* = 30 for the licorice group and *n* = 30 for the triamcinolone group). The study concluded that both licorice and triamcinolone mucoadhesive-based films are effective in reducing oral discomfort in oral mucositis during radiotherapy. Licorice whole extract was also tested for improving the neurological emergency situation in 75 patients suffering from ischemic stroke [173]. Licorice extract was prescribed at the rate of 450 mg or 900 mg capsules for 7 days with a daily frequency of 3 times. In addition, a decline in the National Institute of Health Stroke Scale and also the modified Rankin Scale scores suggest the beneficial effect of licorice extract in improving the neurological condition in acute ischemic stroke patients. In another double-blind clinical trial, licorice roots were evaluated for their ability to relieve hot flashes and their reoccurrence in 90 menopausal women [173]. Consumption of 3 capsules daily containing 330 mg of licorice root extract resulted in a decreased severity and frequency of hot flashes. Taken together, data presented here underline that *Glycyrrhiza* spp. has shown to be effective in reducing the discomfort in cancer, ischemic, neurological, and a few other complications in humans. But, the small number of patients in clinical trials and the very limited trials remain a concern, so that there is a need to pay specific attention in the future to establish *Glycyrrhiza* plant extracts as potential pharma ingredients.

## 7. Toxicological Data, Safety, and Bioavailability Features

Published literature data showed that *Glycyrrhiza glabra* L. is capable of inducing serious adverse effects [174]. Historically, glycyrrhizin is the term used to describe the crude acid extract of licorice. A component of glycyrrhizin is glycyrrhizinic acid that occurs as calcium, potassium, or ammonium salts. Glycyrrhizinic acid consists of 18*β*-glycyrrhetinic acid and 2 glucuronic acid molecules [175].

Generally, the assessment of the pharmacological effects of *Glycyrrhiza glabra* L. are mostly related to its principal components, glycyrrhizin and glycyrrhetinic acid (GA), with both demonstrating positive biological activities, as described in the previous sections [176]. Glycyrrhizinic acid and its salts are hydrolysed by intestinal bacteria and absorbed from the gastrointestinal (GI) tract, with glycyrrhizin hydrolysis being related to specialized *β*-glucuronidase activity [177]. In earlier studies, it was reported that prior to absorption, the microbial hydrolysis of glycyrrhizin leads to the formation of 3*β*-monoglucuronyl-18*β*-glycyrrhetic acid [178], and then GA [179]. Gut microbiota acts differently on glycyrrhizin; some microbial glucuronidases, particularly from *Eubacterium* and *Bacteroides* spp., can fully D-glucuronidate glycyrrhizin, whereas some others from *Streptococcus* spp. strains only remove one glucuronide moiety [180]. For instance, after application of 800 or 1600 mg ammonium salt or licorice extract in healthy adult volunteers, the time to reach maximum plasma concentration of 18*β*-glycyrrhetinic acid was 10 and 12 h, respectively [181]. The interindividual variation in glycyrrhizin response, metabolism, and kinetics is also present, being at least partially caused by the gut microbiota. GA becomes more apparent when the administration occurs on a daily basis, and its complete elimination takes several days [182].

GA is notably absorbed by tissues; however, it greatly adheres to serum albumin [175, 177], with its elimination being directly related to serum protein binding saturation. GA uptake at doses < 10 mg/kg b.w. results in a rapid elimination *via* bile [175]. The plasma clearance of 18*β*-glycyrrhetinic acid is essentially reduced (<38–64 mL/h/kg) in patients with CHC and liver cirrhosis [177]. In special situations, during premenstrual syndrome, the *Glycyrrhiza glabra* L. application may cause water retention and bloating [183].

Regarding glycyrrhizin consumption, it is commonly well-tolerated at low amounts, while at high doses, it may become toxic [184, 185]. In addition, the interaction of glycyrrhizin with other components present in licorice extracts during intestinal intake may lead to modified bioavailability and serious adverse effects [181]. In this regard, a synergistic suppression of NO production and inducible NO synthase expression was observed while using licorice extracts, while the glycyrrhizin treatment alone did not show these effects [186]. Nonetheless, regarding glycyrrhizin, the most important issue seems to be related to the fact that glycyrrhizin suppresses the 11-*β*-hydroxysteroid dehydrogenase type 2 (11-*β*-HSD2) enzyme that leads to high cortisol concentrations in tissues, resulting in inappropriate mineralocorticoid activity with sodium and water retention and resulting in loss of potassium. Thus, because of the long half-life of 18*β*-glycyrrhetinic acid, large doses of KCl supplementation are necessary for weeks [180]. Worthy of note is that this phenomenon has been noted to occur in both humans and animals [187]. Nonetheless, in general, the main adverse effects of *Glycyrrhiza glabra* L. are related to hypertension and various hypokalemic-induced secondary complications (e.g., compensatory reduction in plasma aldosterone and renin concentrations, and hypokalemia) [188]. The summary of the side effects of licorice is presented in Table 1.

Licorice-induced hypokalemia was first described by Revers in 1946 [189]. GA induces pseudohyperaldosteronism by inhibiting 11-*β*-HSD2 [201]. In addition, hypokalemia can result in hypokalemic paralysis, proximal myopathy, rhabdomyolysis, and flaccid quadriparesis [175]. Other reports have also demonstrated other interactions of licorice [202]. Hypokalemia is well-known to aggravate glucose intolerance. Therefore, licorice ingestion may affect blood glucose levels and interfere with hypoglycemic therapy. The licorice component, isoliquiritigenin, prohibits aldose reductase, which reduces glucose to sorbitol, and inhibits sorbitol accumulation in tissues and cells. Nonetheless, patients with apparent mineralocorticoid excess (AME), a rare form of hypertension caused by mutations in the 11-*β*-HSD2 gene, may be responsive to both licorice and its components. It is worth noting that untreated AME may prompt damage to different organs (kidney, colon, salivary glands, and placenta) [203]. On the other hand, individuals may exhibit digoxin toxicity because of hypokalemia induced by licorice absorption [204]. The potentiation of warfarin effects due to the licorice inhibitory effect on the hepatic microsomal enzyme system was also reported [205]. Bilateral median neuropathy in a patient possibly related to licorice-induced water retention was also reported [193]. Other complications include hypersensitivity to glycyrrhizin [206], occupational asthma [207], myoclonus [191], or contact dermatitis [208]. In addition, a number of researches have reported the occurrence of ocular complications related to licorice ingestion [209–212]. Also, individuals with reduced liver function may be extremely vulnerable to an extra use of licorice, as an excessive consumption may cause thrombocytopenia [201]. However, some case statements have clinically proven licorice interactions with drugs. It interacts with different kinds of drugs, namely, steroids that may result in a quick metabolism of the coadministered drugs, *via* induction of different enzymes [213]. For example, the extracts from *Glycyrrhiza glabra* L., *Glycyrrhiza inflata* Batalin, and *Glycyrrhiza uralensis* Fisch. inhibit several drug-metabolizing cytochrome P (CYP) 450 enzymes. In addition, some licorice components, like isoliquiritigenin, licoricidin, licochalcone A, 18*β*-glycyrrhetinic acid, and glycycoumarin, inhibit one or more members of the CYP2C family. CYP1A2, CYP2B6, and CYP3A4 are also inhibited by glycycoumarin and licochalcone A [214].

Although the glycyrrhizin responsiveness is affected by the health status, some patients may show manifestations of toxicity with administration of small doses [215]. Of course, these very sensitive subgroups are comprised of people with decreased 11-*β*-HSD2 activity, or even with a prolonged GI transit [187]. In one of the studies, it was reported that glycyrrhizin and GA bind to nucleic acids. Spectroscopic data have indicated that glycyrrhizin binds to DNA *via* the A-T and PO2 group, with the affinity of ligand-DNA binding of glycyrrhizin being higher than that of GA [216, 217]. In turn, RNA is binding *via* the G-C and A-U base pairs, with the affinity of ligand-RNA binding being in the same order [218].

*Glycyrrhiza glabra* L. crude extracts also have estrogenic effects [219]. The obtained data have demonstrated that *Glycyrrhiza* spp. have different zones of estrogenic action, and this underlined the need for precise labeling of herbal supplements. The application of licorice was demonstrated by a rise in blood pressure of pregnant women [220]. Moreover, in another study, the administration of ammonium glycyrrhizinate to rats from 7 to 17 days of pregnancy led to a rise in the prevalence of external hemorrhages and hematomas [221]. Also, high glycyrrhizin doses did not notably affect the birth weight or maternal blood pressure [222], despite that high glycyrrhizin exposure was correlated with shorter gestational period, raising more than two times the risk of preterm (<37 weeks) delivery [223]. Other authors also stated that when children age reaches 8 years, they have poorer cognitive completion, externalizing symptoms and attention issues after high glycyrrhizin exposure [224]. At the age of 12 years, girls, but not boys, were taller, heavier, and had higher body mass index for age [225]. Moreover, children scored lower on tests of intelligence quotient, had faulty memory, and had higher odds of attention deficit/hyperactivity disorder. The outcome in these investigations indicated the potential adverse action during pregnancy. Based on some findings, the negative health effects of glycyrrhizin administration in mothers or their fetus or child were found at doses ≥ 500 mg/week [222–226]. Nonetheless, due to the various adverse effects reported, complex well-designed human trials of pregnant women with accurately estimated exposure are needed [227, 228].

The basic problem with licorice dosing lies in its availability in diverse forms and at distinct doses. The combination of a long persistence and enterohepatic circulation along with the unreliable bioavailability has made difficulty in establishing a clear dose-response correlation for glycyrrhizinate substances. Another issue found is the high interindividual variation in sensitivity to glycyrrhizin and glycyrrhizic acid. Many possible reasons for this fact exist; however, the main reason seems to be related to differences in the gut microbiota ability to hydrolyze glycyrrhizic acid to glycyrrhetic acid [177]. Rare causes of apparent mineralocorticoid excess include the genetic deficiency of 11-*β*-HSD2, sometimes present in adulthood, which although usually appears in childhood, it is often asymptomatic and may stay undiagnosed until adulthood [229]. An *in vitro* evaluation demonstrated that glycyrrhetinic acid in humans at relevant concentrations may disturb cell adhesion, effect anoikis-like cell death, cause morphologic changes, and disturb cytoskeletal proteins [230].

The Food and Drug Administration (FDA) declared licorice root extracts and ammoniated glycyrrhizin as generally recognized as safe (GRAS). However, glycyrrhizinic acid is used for the production of many additive agents in the USA at the following maximum permitted levels: baked foods = 500 mg/kg; alcoholic beverages = 1000 mg/kg; nonalcoholic beverages = 1500 mg/kg; chewing gum = 11000 mg/kg; hard candy = 160000 mg/kg; herbs and seasonings = 1500 mg/kg; plant protein products = 1500 mg/kg; soft candy = 31000 mg/kg; and vitamin or mineral dietary supplements = 5000 mg/kg. The maximum levels of glycyrrhizin allowed in food products are 16% for hard candy and 3.1% for soft candy. The maximum allowable levels in other foods ranged from 0.05 to 0.15% [231].

The Flavor and Extract Manufacturers Association (FEMA) has noted the following maximum levels of glycyrrhizic acid ammonium salt: 51 mg/kg for nonalcoholic beverages; 59 mg/kg for alcoholic beverages; 61 mg/kg for baked goods; 79 mg/kg for gelatin/puddings; 91 mg/kg for frozen dairy products; 625 mg/kg for frosting confectionery; 676 mg/kg for hard candies; 1511 mg/kg for soft candies; and 2278 mg/kg for chewing gum [177]. The safe levels of glycyrrhizic acid and its salt are given in Table 2.

However, according to the FAO/WHO Committee, the administration of 80–100 mg glycyrrhizic acid daily was referred to as being capable of provoking hypertension, so that the safety evaluation of glycyrrhizinic acid should be based on human data [175]. In Europe, the European Medicines Agency (EMA) concluded that there are no clinical data in the research-based report to support a “well-established medicinal use.” Although the short-term application (≤4–6 weeks) of licorice preparations was judged as safe, there is insufficient data to support licorice root safety during pregnancy and lactation, and in children and adolescents (<18 years). So, the use is not recommended for these groups [213]. The European Food Safety Authority (EFSA) panel decided that licorice extract as a food additive is safe for the main human adult population up to 100 mg daily [232]. Taken together, all data presented above indicate that licorice is safe to consume for maintaining human health, but the data also reinforced the need of a precise labeling of botanical supplements containing licorice compounds.

## 8. Conclusions

*Glycyrrhiza* spp. has been used for food and medicinal purposes around the world from ancient times. It is conspicuous that licorice rhizomes and roots have been widely used in many countries, with its medicinal properties being mostly conferred by phytoconstituents. Glycyrrhizin along with liquiritigenin, isoliquiritigenin, 4′-O-methylglabridin, isoprenyl chalcone, formononetin, glabridin, and hispaglabridins A and B are the main phytochemicals present in *Glycyrrhiza* spp., although many new compounds have been progressively discovered. Both the isolated compounds and extracts from *Glycyrrhiza* spp. have shown key antioxidant, antimicrobial, anti-inflammatory, antiproliferative, and cytotoxic effects through *in vivo* and *in vitro* experiments. The biological activities of glycyrrhizin (glycyrrhizic acid) and *Glycyrrhiza glabra* L. are the most evaluated compound and plant species. Clinical trials based on *Glycyrrhiza* spp. have established that its extracts are useful in reducing the issues of discomfort in cancer, ischemic, and neurological patients. For continuing work on licorice, more in-depth studies are needed to clarify some gaps related to its safety and toxicological features along with clinical trials that are needed for establishing *Glycyrrhiza* plant extracts as potential pharma and food ingredients.

## Figures and Tables

**Table 1:** Typical examples of *Glycyrrhiza glabra* L. and glycyrrhizin salt-related side effects in humans.

Gender	Age (in years)	Consumption	Symptoms/complications	Reference
Man	70	Licorice candies, 60–100 g/daily for 4–5 years	Hypertension, hypokalemia, rhabdomyolysis	[190]
Woman	90	Antacid with licorice	Hypertension, hypokalemia, myoclonus, metabolic alkalosis	[191]
Woman	55	High amount of licorice, daily, for 4 years	Hypertension, hypokalemia	[192]
Woman	44	Chewing licorice sticks, 3 days	Edema, bilateral nocturnal hand pain, paresthesias in fingers, bilateral carpal tunnel syndrome	[193]
Woman	46	Herbal tea with licorice, 1-2 cups/daily for 7 years	Hypertension, hypokalemia, reduced plasma aldosterone, and renin	[194]
Woman	39	Herbal medicine with licorice/daily for 8 weeks	Hypertension, hypokalemia, muscle weakness, acute renal failure	[195]
Woman	38	Licorice root tea, 3 times per day for 2 months	Polymorphic, ventricular tachycardia	[196]
Man	52	Licorice, 1.5 g/daily for 2 months	Hypertension, severe asthenia, muscle cramps	[197]
Man	49	Herbal formulation containing licorice, for 1.5 years	Hypokalemia, somnolence, rhabdomyolysis, acute renal failure	[198]
Man	57	Licorice, 900 g/weekly for 3–4 months	Hypertension, acute visual impairment, hypokalemia	[199]
Man	72	Licorice, 2 oz/weekly for 1 month	Hypertension, metabolic alkalosis, severe hypokalemia, increase in rhabdomyolysis indexes myoglobinuria	[200]

**Table 2:** Summary of suggested safe levels of glycyrrhizic acid and its salts.

Recommended safe levels	Source
GRAS	[231]
100 mg daily	[233]
100 mg daily	[175, 234]
Not possible to establish	[213]
100 mg daily	[232]
